# Assessment of Vitamin B_12_ and Its Correlation with Dental Caries and Gingival Diseases in 10- to 14-year-old Children: A Cross-sectional Study

**DOI:** 10.5005/jp-journals-10005-1424

**Published:** 2017-06-01

**Authors:** Shivayogi M Hugar, Neha S Dhariwal, Andleeb Majeed, Chandrashekhar Badakar, Niraj Gokhale, Laresh Mistry

**Affiliations:** 1 Professor and Head, Department of Pedodontics and Preventive Dentistry VK Institute of Dental Sciences, KLE University, Belagavi Karnataka, India; 2 Postgraduate Student, Department of Pedodontics and Preventive Dentistry VK Institute of Dental Sciences, KLE University, Belagavi Karnataka, India; 3 Postgraduate Student, Department of Pediatrics, Jawaharlal Nehru Medical College KLE University, Belagavi, Karnataka, India; 4 Reader, Department of Pedodontics and Preventive Dentistry VK Institute of Dental Sciences, KLE University, Belagavi Karnataka, India; 5 Lecturer, Department of Pedodontics and Preventive Dentistry VK Institute of Dental Sciences, KLE University, Belagavi Karnataka, India; 6 Postgraduate Student, Department of Pedodontics and Preventive Dentistry VK Institute of Dental Sciences, KLE University, Belagavi Karnataka, India

**Keywords:** Dental caries, Oral health, Vitamin B_12_.

## Abstract

**Aim:**

To assess the level of vitamin B_12_ and correlate it with dental caries [decayed, missing, and filled permanent teeth (DMFT) score] and gingival diseases [plaque index (PI) and gingival index (GI)].

**Design:**

Healthy children according to the inclusion criteria were selected by the computerized randomization method from a school to assess the vitamin B_12_ levels using Centaur/ Versace machine.

**Materials and methods:**

Blood samples were collected to assess vitamin B_12_ levels using automated analyzer. Oral examination was done by a single calibrated dentist. A thorough oral examination was carried out and the DMFT, PI, and GI scores of all the children were recorded and assessed. Data were analyzed using Karl Pearson’s correlation test.

**Results:**

Vitamin B_12_ levels were deficient in 64% of the children. In boys, vitamin B_12_ deficiency was found in about 76.2%, whereas, in girls it was 57.1%, which was not statistically significant. The vitamin B_12_ deficient children showed a significantly high DMFT scores than the children with normal vitamin B_12_ levels. The Pearson’s correlation was -0.614 for DMFT, PI value -0.663, and GI value of -0.477. The negative correlation stated that there was a reverse relation between these indices and vitamin B_12_.

**Conclusion:**

In children with systemic vitamin B_12_ deficiency, there is increased dental caries prevalence and associated gingival problems.

**How to cite this article:**

Hugar SM, Dhariwal NS, Majeed A, Badakar C, Gokhale N, Mistry L. Assessment of Vitamin B_12 _and Its Correlation with Dental Caries and Gingival Diseases in 10- to 14-year-old Children: A Cross-sectional Study. Int J Clin Pediatr Dent 2017;10(2):142-146.

## INTRODUCTION

Vitamin B_12_ is one of the important micronutrients for brain development and function. The developing brain was more sensitive to the deficiency of this micronutrient than the mature brain. Fetal requirements are obtained by active transport through the placenta. Vitamin B_12 _(cobalamin) deficiency is common in Indians; largely owing to vegetarianism.^1^

Micronutrient deficiency is a serious childhood problem in developing countries. Deficiencies of vitamins A, B_12_, iron, folic acid, and zinc are preventable causes of poor child growth and school performance.^2^ Evidence regarding B_12_ deficiency, it’s incidence is unknown in India, however, recent studies suggests that it is commoner than thought. Large number of cases were reported with neurological manifestations. Studies by Wadia et al^3^ found vitamin B_12_ deficiency (below 200 pg/mL) in 0.88%, folic acid deficiency in 1.1% (below 3 ng/mL), deficiency of both in 2.4% patient, and 3.8% had levels near the lower limit of normal.^3^ It is common in India, owing to cultural and religious beliefs, low intake of animal source food, and strict vegetarianism.^1,4,5^

The consequences of cobalamin deficiency include poor growth, megaloblastic anemia, neurological manifestations include mood changes and altered sensory, and motor and cognitive functions.^6^

For the pediatric dentists, it is a matter of concern because oral health is the reflection of the body. Healthy teeth and gums provide a healthy body. Poor oral health has its effects to various organ systems, during pregnancy causing prematurity to long-term effects in off-springs, can be a risk factor to heart diseases. There are certain essential nutrients, micro- and macro-nutrients which are required for overall development of an individual.^7,8^

Vitamin B_12_ is found as cobalamin only in animal products. Many poor population or those who avoid animal products for religion or other reasons consume little or no Vitamin B_12_. Low serum B_12_ concentration is associated with a higher risk of harm to short-term memory, cognitive function, and higher risk of megalo-blastic anemia.^2,9-11^

Cobalamin deficiency is inadequate to support normal metabolic function.^12^ Inadequate intakes of nutrients, e.g., riboflavin, copper, vitamin D, and vitamin B_12_, are associated with increased caries experience.^13^

Thus, in this study, an attempt is made to assess the level of vitamin B_12_ and correlate it with dental caries and gingival diseases.

## MATERIALS AND METHODS

The protocol of the present study was approved by the ethical committee and scientific review board of the university. The study comprised of 42 children in the age group of 10 to 14 years (n = 21 girls and n = 21 boys). The children were selected randomly according to the inclusion criteria. Children who were healthy and compliant were selected. Children with any systemic illness or problems excluded from the study. Also, the patients not willing to accept proposed treatment plan and/or participate in the study were not included. Informed written consent was obtained from all the parents of children participating in the study. Assent was obtained from all the children participating in the study. After recording the preliminary information, clinical examination was carried out.

## METHODOLOGY

All the adolescent children between the age group of 10 to 14 years (21 girls and 21 boys) that fit into the study were selected by computerized randomization method and screened for vitamin B_12_ deficiency. Blood samples were collected by pathologist. Vitamin B_12_ levels were assessed by Centaur/Versace machine and hemoglobin levels by BEKMANN and COULTER LH 500 automated hematology analyzer.

### Sample Collection and Storage

Serum

A serum separator tube was used and 3cc blood samples were allowed to clot for 2 hours at room temperature or overnight at 4°C before centrifugation for 20 minutes at approximately 1000 gm. Freshly prepared assay serum was immediately stored in aliquot at -20°C for later use. Repeated freeze/thaw cycles were avoided.

Plasma

Plasma was collected using Ethylenediaminetetraacetic acid or heparin as an anticoagulant. Samples were cen-trifuged for 15 minutes at 1000 gm at 2 to 8°C within 30 minutes of collection. Plasma and assay were removed immediately or samples were stored in aliquot at -20°C for later use. Repeated freeze/thaw cycles were avoided.

Oral Examination

Oral evaluation was done for all the children selected for the study. Before doing the oral examination, diet history and other relevant details if patient is under any vitamin or calcium supplements was noted.

Oral examination of all children, along with clinical examination of teeth and periodontal tissues were done by the same calibrated dentist. The visual examination was done under standard dental chair and light. Intraoral illumination was provided by a headlamp with the aid of an ordinary mouth mirror. A dental explorer was used for caries detection and gingival examination. All the teeth surfaces were dried and visualized. Scores for decayed, missing, and filled permanent teeth (DMFT) was done to assess the caries prevalence according to World Health Organization criteria.

Gingival health and oral hygiene scores were assessed using the gingival index (GI) (Lüe and Silness, 1963) and plaque index (PI) (Silness and Lüe, 1964). The GI scores are as follows: 0 = absence of inflammation/normal gingiva; 1 = mild inflammation, slight change in color, slight edema, and no bleeding on probing; 2 = moderate inflammation, moderate glazing, redness, edema, and hypertrophy; bleeding on probing; 3 = severe inflammation, marked redness, and hypertrophy ulceration and tendency to spontaneous bleeding. The PI scores are as follows: 0 = no plaque; 1 = a film of plaque adhering to the free gingival margin and adjacent area of the tooth. It can be seen by using a probe on the tooth surface; 2 = moderate accumulation of soft deposits within the gingival pocket, or the tooth and gingival margin which can be seen with the naked eye; 3 = abundance of soft matter within the gingival pocket or on the tooth and gingival margin. The dental examination was done by one examiner only. The data were statistically analyzed by using Statistical Package for the Social Sciences 18 software. Karl Pearson’s correlation method was used to find out the relation between the DMFT, GI, and PI and the level of vitamin B_12_. P-value less than 0.05 was considered significant.

**Graph 1:**
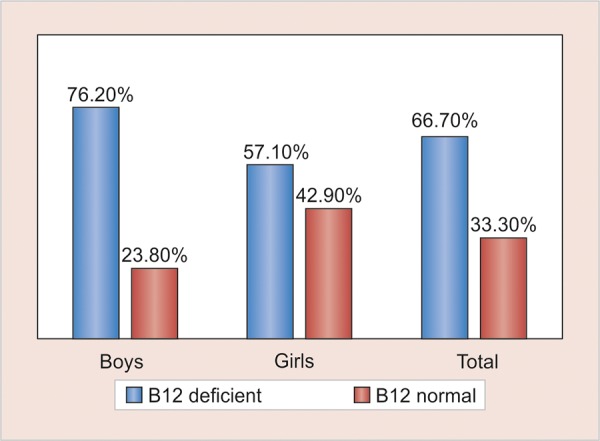
Gender distribution and level of vitamin B_12_

**Graph 2:**
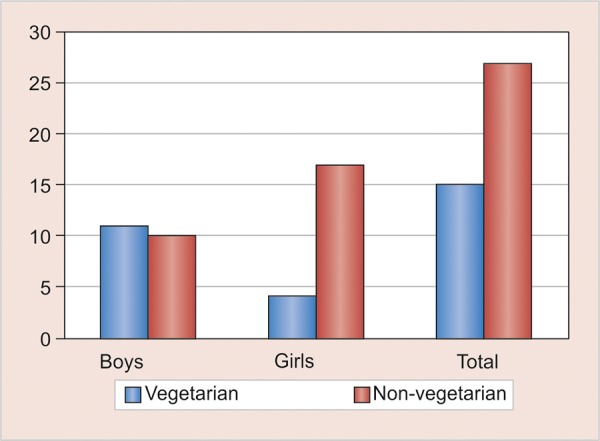
Gender and the diet distribution

*Results:* There were equal number of girls and boys in the present study. The case history revealed all the children had similar oral hygiene practices and did not have any other systemic problems or were not under any medication. The vitamin B_12_ levels were deficient in 64% of the children (Graph 1). In boys, vitamin B_12_ deficiency was found in about 76.2%, whereas, in girls it was 57.1% (Graph 1) which was not statistically significant. In the diet and gender distribution, 52.4% boys had a vegetarian diet, whereas only 19% girls had a vegetarian diet (Graph 2). The mean DMFT score was 3.810, PI score was 1.376, and GI score was 0.914. The vitamin B_12_ deficient children showed a significantly high DMFT scores than the children with normal vitamin B_12_ levels. The Pearson’s correlation being -0.614 for DMFT, PI value of -0.663, and GI value of -0.477. In Tables 1 to 3, the diet and dental caries relation also showed that the dental caries was higher among the vegetarian diet as compared to the non-vegetarian diet (Tables 4 and 5). The negative correlation stated that there was a reverse relation between these indices and vitamin B_12_ stating that when the vitamin B_12_ is deficient, there increase in dental caries and gingival problems.

**Table 1:** **Table 1:** Correlation between dental caries and vitamin B_12 _levels using Karl Pearson’s correlation

				*DMFT*		*Vitamin B_12_*	
DMFT		Pearson’s correlation		1		–0.614**	
		Sig. (2-tailed)				0.000	
		N		42		42	
Vitamin B_12_		Pearson’s correlation		–0.614**		1	
		Sig. (2-tailed)		0.000			
		N		42		42	

**Correlation is significant at the 0.01 level (2-tailed)

**Table 2:** **Table 2:** Correlation between plaque index and vitamin B_12 _levels using Karl Pearson’s correlation

				*PI*		*Vitamin* B_12_	
PI		Pearson’s correlation		1		–0.663**	
		Sig. (2-tailed)				0.000	
		N		42		42	
Vitamin B_12_		Pearson’s correlation		–0.663**		1	
		Sig. (2-tailed)		0.000			
		N		42		42	

**Correlation is significant at the 0.01 level (2-tailed)

**Table 3:** **Table 3:** Correlation between gingival index and vitamin B_12 _levels using Karl Pearson’s correlation

				*Vitamin* B_12_		*GI*	
Vitamin B_12_		Pearson’s correlation		1		–0.477**	
		Sig. (2-tailed)				0.001	
		N		42		42	
GI		Pearson’s correlation		–0.477**		1	
		Sig. (2-tailed)		0.001			
				42		42	

**Table 4:** **Table 4:** Correlation between diet, B_12_ deficiency, and DMFT scores

*Mean DMFT score*							
*Vegetarian*				*Non-vegetarian*			
Deficient B_12_		Normal B_12_		B_12_ deficient		Normal B_12_	
5.4		1.4		4.08		1.7	

**Table 5:** **Table 5:** Correlation between diet and vitamin B_12_ levels using Karl Pearson’s correlation

						*Vitamin* B_12_						*Average DMFT*	
						*Deficient*		*Normal*		*Total*		*scores*	
Diet		Vegetarian		Count		10		5		15		4.5	
				% within diet		66.7%		33.3%		100.0%			
				% of total		23.8%		11.9%		35.7%			
		Non-vegetarian		Count		18		9		27		3.04	
				% within diet		66.7%		33.3%		100.0%			
				% of total		42.9%		21.4%		64.3%			
Total				Count		28		14		42			
				% within diet		66.7%		33.3%		100.0%			
				% of total		66.7%		33.3%		100.0%			

## DISCUSSION

Many elements in trace amounts are known to contribute to the oral hygiene and health.^14^ Vitamin B_12_ is an essential element which is not produced by the body. It is mainly found exclusively in animal products, such as meat, eggs, fish, minute quantities in milk, soy, etc.^15^ It is important in the early growth of the child as it affects brain development and function, memory, reasoning, attention, metabolism, formation of RBC’s, and oral hygiene as stated by various authors.^12,15,16^ Inadequate intakes of nutrients (e.g., riboflavin, copper, vitamin D, and vitamin B_12_) associated with increased caries experience. Adequate intake *vs* inadequate or high adequate intakes of nutrients (e.g., vitamin B_12_ and vitamin C) were associated with decreased caries experience.^13^

Vitamin B_12_ is one of the most common deficiencies in the Indian population, however, most often undetected and found as an accidental finding. The relationship between vitamin B_12_ deficiency and oral health still remains unclear. The available medical literature does not show any study performed with such a correlation. Since various studies have stated that the oral conditions differ from geographic and social conditions. The children going to the same school and having similar dental care habits and characteristics were selected for this study. Also other systemic illnesses or any calcium supplements or medications that would hamper the oral hygiene were taken into consideration to eliminate any false results or bias. The method used for vitamin B_12_ assessment is the most commonly used and also standard as stated in various studies.

The DMFT/decayed, missing and filled surface indices are the most commonly used tools to determine the caries prevalence and health status of the populations. The GI and PI scores are the gold standards for examining the oral hygiene status of the subjects. In this study, the overall effect of vitamin B_12_ deficiency was measured together on the teeth and gingiva.

There is not much data available in this regard. Mar-shall^13^ found that inadequate intakes *vs* low adequate or high adequate intakes) of nutrients (e.g., riboflavin, copper, vitamin D, and vitamin B_12_) were associated with increased caries experience and low adequate intakes *vs* inadequate or high adequate intakes) of nutrients (e.g., vitamin B_12_ and vitamin C) were associated with decreased caries experience.

Pontes et al^11^ also found presence of oral signs and symptoms, including glossitis, angular cheilitis, recurrent oral ulcer, oral candidiasis, diffuse erythematous muco-sitis, and pale oral mucosa in subjects with cobalamin deficiency offering the dentist an opportunity to participate in the diagnosis of this condition. Supplementation with vitamin B_12_ will improve the gingival health and oral hygiene of children with deficiency, the present study was only a cross-sectional and the changes in the oral health could not be demonstrated, which will be further explored in further studies. Also an in-depth salivary analysis to rule out any other causes with a larger sample size will further validate the results of the present study.

The presence of statistically significant results with a reverse relation makes a good evidence and logic for the association between them. As pediatric dentists, we must be aware of the possible deficiency taking into consideration the history, clinical signs, and also investigations whenever required. This will prevent the further progress of the disease and any irreversible damage to the neural and cognitive function.

## CONCLUSION

Vitamin B_12_ deficiency may cause an increase in prevalence of dental caries and gingival diseases in children. As pediatric dentist, our role is not just giving dental care to the child but it also includes the overall health care, behavior assessment which will provide us with the sign and symptoms of any deficiency or abnormality in child and with timely referral to pediatrician and concern of the dentist, we can prevent any permanent damage to the children.
